# Eye Movement Parameters for Performance Evaluation in Projection-based Stereoscopic Display

**DOI:** 10.16910/jemr.11.6.3

**Published:** 2018-11-20

**Authors:** Chiuhsiang Joe Lin, Yogi Tri Prasetyo, Retno Widyaningrum

**Affiliations:** Department of Industrial Management, National Taiwan University of Science and Technology, Taiwan; Department of Industrial Engineering Sepuluh Nopember Institute of Technology, Kampus ITS Sukolilo Surabaya 60111, Indonesia

**Keywords:** Structural equation modeling, mediator effect, eye movement parameters, stereoscopic, parallax, virtual reality, eye movement, eye tracking

## Abstract

The current study applied Structural Equation Modeling (SEM) to analyze the rela-tionship among index of difficulty (ID) and parallax on eye gaze movement time (EMT), fixation duration (FD), time to first fixation (TFF), number of fixation (NF), and eye gaze accuracy (AC) simultaneously. EMT, FD, TFF, NF, and AC were measured in the projec-tion-based stereoscopic display by utilizing Tobii eye tracker system. Ten participants were recruited to perform multi-directional tapping task using within-subject design with three different levels of parallax and six different levels of ID. SEM proved that ID had significant direct effects on EMT, NF, and FD also a significant indirect effect on NF. However, ID was found not a strong predictor for AC. SEM also proved that parallax had significant direct effects on EMT, NF, FD, TFF, and AC. Apart from the direct effect, parallax also had significant indirect effects on NF and AC. Regarding the interrelation-ship among dependent variables, there were significant indirect effects of FD and TFF on AC. Our results concluded that higher AC was achieved by lowering parallax (at the screen), longer EMT, higher NF, longer FD, and longer TFF.

**Practitioner Summary**: The SEM could provide valuable theoretical foundations of the interrelationship among eye movement parameters for VR researchers and human-virtual-reality interface developers especially for predicting eye gaze accuracy.

## Introduction

Virtual reality (VR) has developed significantly in the world over the past two decades. It is designed to make possible a human sensorimotor and cognitive activity in a digitally created artificial world, which can be imaginary, symbolic, or a simulation of certain aspects of the real world (1). Manufacturers and researchers from different disciplines are paying more and more attention to VR, seeking to maximize the image quality while also considering the diverse applications. Recent research has explored the promising diverse applications of VR, particularly in the 3D geovisualization (2), 3D animated media (3), and even 3D laparoscopic surgery (4). One of the most common techniques to create VR is projection-based stereoscopic display.

Projection-based stereoscopic display has been commercialized in order to implement it in VR (5). It generates 3D images by creating depth perception via a cue called binocular disparity which refers to a lateral shift or difference between the spatial positions of corresponding left and right eye images (6). This binocular disparity of two images between left and right eye is commonly mentioned as parallax (7). Parallax creates binocular disparity in the human visual system that gives a stereoscopic effect of depth with each eye receiving an image similar, but not identical, to that of a real spatial vision (1). One common device to evaluate the effectiveness of projection-based stereoscopic display is eye tracker (8, 9)

Eye tracker is becoming widely popular to evaluate projection-based stereoscopic 3D display, especially for collecting and analyzing information about the users. It is a tool that allows user experience researchers to observe the position of the eye to understand area of interest an individual is looking (10). Eye tracker measures some variables which commonly named as eye movement measures or eye movement parameters. Research in different fields might focus on different eye movement parameters (11).

**Fig. 1.**
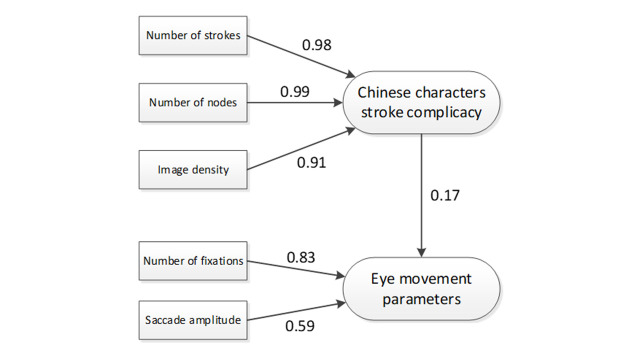
Structural Equation Modeling of Chinese character complicacy using eye movement parameters (12).

Despite the availability of many eye tracker publications over the past two decades, there is still little information available on the interrelationship among eye movement parameters. Previously, Ma & Chuang (38) investigated the correlations between the Chinese characters stroke complicacy and eye movement parameters by utilizing structural equation modeling (SEM). However, the path coefficient was found low (β: 0.17) and the loading factor of saccade amplitude was less than 0.70 (p-value < 0.10) indicating that saccade amplitude was a not a strong predictor for eye movement parameters in the model (Figure 1). Moreover, the interrelationship between two eye movement parameters (NF and saccade amplitude) and Chinese characters information was not analyzed further. Unema et al (13) mentioned that there was a strong but nonlinear relationship between saccade amplitude and fixation duration. Similarly, Pannash et al (14) demonstrate a systematic change in the saccade amplitude and fixation duration over time. However, these studies were limited only to two eye movement parameters. A further investigation which incorporates more eye movement parameters could be very valuable for VR researchers on different fields and human-virtual reality interface developers. Goldberg conducted a study to investigate the impact of several page design factors on perceived ratings of page clarity, completion time, emotional valence from video, and several eye movement parameters (15). In addition, Goldberg also explored the relationship among selected eye movement parameters using Pearson correlation (Table 1) (15). This study could be improved by utilizing SEM approach since this method can analyze beyond a simple correlation analysis.

**Table 1.** Correlation matrix among selected eye movement parameters (15).

	SO	JF	SR	CT	EV	TFF	FD	NF
JF	.17***							
SR	.21***	ns						
CT	.16***	.10*	.45***					
EV	ns	ns	ns	ns				
TFF	.15**	.24***	.23***	.43***	ns			
FD	.14**	ns	ns	ns	ns	ns		
NF	.25***	ns	.41***	.82***	ns	.40***	ns	
SA	ns	ns	.21***	.30***	ns	.41***	ns	.35***

*Note:* JF=JPEG file size; SR=subjective ratings; CT=task completion time; EV=emotional valence; TFF=time to first fixation; FD=fixation duration; NF=number of fixations; SA=search area. *p<0.05. **p<0.01. ***p<0.001.

SEM is a very useful technique to investigate the interrelationship among eye movement parameters since the relationships between variables are assessed simultaneously via covariance analysis (16). It examines the structure of interrelationships expressed in a series of equations, similar to a series of multiple regression (17). A structural model with a hypothesized mediating effect can also produce direct and indirect effects (17). Direct effects are the relationship linking two parameters with a single path and indirect effects are those relationships that involve a sequence of relationships with at least one intervening parameter involved (17). Two of our previous studies collected several eye movement parameters in projection-based stereoscopic display which consist of eye gaze movement time (EMT), fixation duration (FD), time to first fixation (TFF), number of fixation (NF), and eye gaze accuracy (AC) (8, 9). Our previous one-way repeated ANOVA analysis must decompose chains of relationships among three or more constructs into tests of relationships to derive the mediating effects. Moreover, our previous one-way repeated ANOVA analysis could not investigate further the interrelationship among dependent eye movement parameters. By utilizing SEM approach, the mediating effects when the third parameter intervenes between two other related parameters and the interrelationship among eye movement parameters can be analyzed simultaneously.

The purpose of the current study is to analyze the interrelationship among eye movement parameters in projection-based stereoscopic display by utilizing SEM approach. The interrelationship among variables could be used to predict AC, which was defined as the distance between the recorded fixation locations and the actual location of the projection of the image (9). In terms of engineering application, AC is one of the most important eye movement parameters and commonly used as the performance evaluation of eye tracker since it can be an objective indicator to distinguish good and bad designs (9, 18, 19). AC could also provide valuable theoretical foundations for VR researchers and human-virtual reality interface developers.

**Fig. 2.**
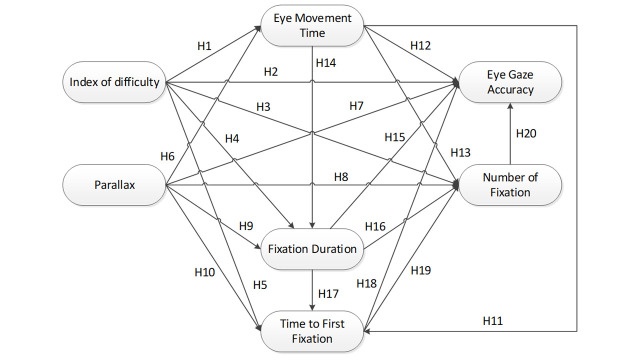
The SEM hypothesis constructs for eye movement parameters in stereoscopic display.

For the hypothesized SEM model, the current study proposed 20 set of hypotheses (Figure 2). ID was hypothesized had significant direct effects on EMT (Hypothesis 1), AC (Hypothesis 2), NF (Hypothesis 3), FD (Hypothesis 4), and TFF (Hypothesis 5). Hypothesis 4 was supported by Walshe & Nuthmann who mentioned that FD was found to be under direct control of stimulus content (20). Following our two previous publications regarding parallax effect on eye movement parameters in stereoscopic display (8, 9), parallax was hypothesized had significant direct effects on EMT (Hypothesis 6), AC (Hypothesis 7), NF (Hypothesis 8), FD (Hypothesis 9), and TFF (Hypothesis 10). EMT was hypothesized had direct effects on TFF (Hypothesis 11), NF (Hypothesis 13), and FD (Hypothesis 14). Based speed-accuracy trade-off in Fit’s Law, EMT was also hypothesized had a significant direct effect on AC (Hypothesis 12). Rodrigues & Rosa., (11) and Castner & Eastman (21) mentioned that FD is highly correlated with NF, therefore FD was hypothesized had a direct effect on NF (Hypothesis 16). FD was also hypothesized had significant direct effects on AC (Hypothesis 15) and TFF (Hypothesis 17). TFF was hypothesized had significant direct effects on AC (Hypothesis 18) and NF (Hypothesis 19) as supported by Goldberg, (2014). Finally, NF was hypothesized had a significant effect on AC (Hypothesis 20) as supported by Togami (22).

## Methods

The current study applied Structural Equation Modeling (SEM) to analyze the interrelationship among index of difficulty (ID), parallax, and eye movement parameters which include eye gaze movement time (EMT), fixation duration (FD), time to first fixation (TFF), number of fixation (NF), and eye gaze accuracy (AC) simultaneously. The main focus of the study is to analyze the causal relationship among all of the parameters for predicting AC.

### Participants

A total of ten participants (7 male and 3 female) from National Taiwan University of Science and Technology voluntary took part in this experiment. Therefore, they were not paid or compensated with academic credits. All participants were graduate students (mean: 25 years; sd: 4 years) and had normal or corrected to normal visual acuity (1.0 in decimal units). Prior to the experiment, participants needed to fill out a consent form and screened for capability to see the object clearly in the stereoscopic display.

### Apparatus

Eye movements were recorded using the Tobii X2-60 remote eye tracking system at a sampling rate of 60 Hz. The fixation filter Tobii Studio version 3.3.2 was used for calibration, testing, and data analysis. Raw eye fixation data was filtered using an I-VT fixation filter with 30 degree per second velocity threshold.

During the experiment, participants were asked to wear a pair of View Sonic 3D glasses PDF-250 to perceive the stereoscopic 3D environment. The 3D glasses were integrated with a 3D vision IR Emitter NVIDIA and 3D View Sonic (PJD 6251) projector. 3D vision IR Emitter NVIDIA was located under the table on a certain distance from Tobii X2 to eliminate the shuttering effect so the signal of 3D Emitter NVIDIA and infrared from Tobii X2 did not affect the eye movement registration. The length and width of the projection screen were 143 x 108 cm respectively. In addition, a Logitech C-920 webcam integrated with Tobii studio was used to record the eye movement data from the screen display.

**Fig. 3.**
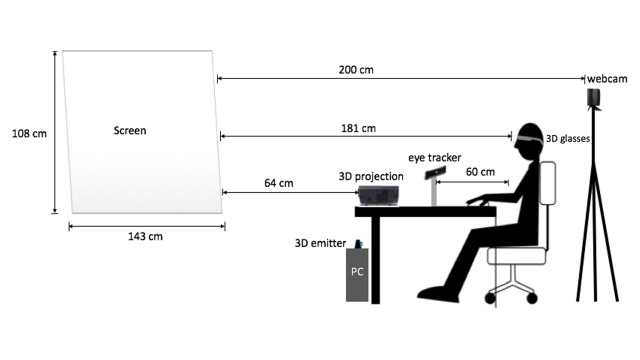
An illustration of experimental layout of the current study (9).

Figure 3 represents the experimental layout of this study. The distance between the participant and the screen was 181 cm. The View Sonic 3D projector was placed 89 in front of the screen and the Tobii eye tracker was placed 64 cm in front of the participant. To maintain the consistency of the relative distance to the participant, all devices were kept fixed and marked using adhesive tape. The participant performed the entire task in a dark room (3.6x3.2x2.5m) covered by black curtains to prevent the light and create a good quality of the stereoscopic environment.

### Independent variables

There were two independent variables in the current study: ID and parallax. ID represents task difficulty and precision level determined by movement distance and object width during tapping task (23). ISO 9241-9 classified the task precision into three levels to measure the accuracy for tapping task: low, medium, and high (24). Table 2 shows the details of ID and task precision level which was similar to our previous study (9). Parallax represents the horizontal display disparity of two images between right and left eyes to create 3D images (7). When the object observed is located virtually in front of the screen, the parallax is negative (1). In the current study, we developed zero parallax (at the screen), negative parallax 20 cm, and negative parallax 50 cm in front of the screen (9). This range was selected to minimize the effect of visual fatigue (8). Since there were six levels of ID and three levels of parallax, therefore there were 18 different combinations need to be completed by the participants.

**Table 2.** ID and task precision level

Distance (unity unit)	Width (unity unit)	ID (bits)	Task Precision Level
20	3.3	2.8	Low
20	2.3	3.3	Low
20	0.6	5.1	Medium
40	3.3	3.7	Low
40	2.3	4.2	Medium
40	0.6	6.1	High

**Table 3.**
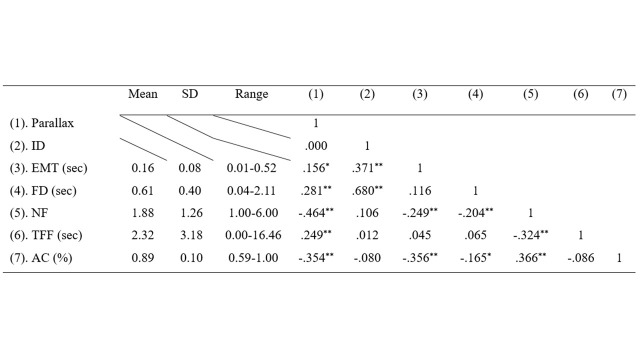
Descriptive statistics and correlation coefficients among the observed variables.

### Dependent variables

There were five independent variables in the current study: eye gaze movement time (EMT), fixation duration (FD), time to first fixation (TFF), number of fixation (NF), and eye gaze accuracy (AC). EMT was the elapsed time from the eye fixation point on the origin to the fixation point of the next target (8). FD or average fixation duration (25) was defined as an average duration of fixations made by the participant to click the virtual target from the origin to the next target. TFF was elapsed time from the slide presentation until the first fixation on the virtual target (15). NF is a total number of fixations counted starting from the origin virtual to destination virtual ball. As mentioned in the introduction, AC was defined as the distance between the recorded fixation locations and the actual location of the projection of the image (9). Following our previous publication (9), AC was calculated using the following formula:

**Figure:**
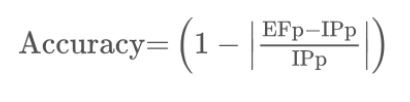


Where,

EFp = Eye fixation position

IPp = Image projection position

Eye fixation positions (EFp) were recorded by utilizing Tobii studio in pixels, and the coordinate positions were converted into mm (8). The image projection position (IPp) was measured from the location of the projection image to the screen in mm. Both EFp and IPp were measured in X-axis and Y-axis (2D). The y-axis was measured from the bottom to the top and the x-axis was measured from left to right. We did not measure the AC in the z-axis. As an independent variable (parallax), we manipulated z-axis into three different levels: 0cm, 20cm, and 50cm in front of the screen. The detailed calculation of EMT had been published in Lin & Widyaningrum (8) and the detailed calculation of FD, TFF, and NF had been published in Lin & Widyaningrum (8). Table 3 represents the descriptive statistics and Pearson correlation coefficients among the observed variables as recommended by (15).

### Experiment Procedures

The experiment was conducted according to the ethical guidelines of the National Taiwan University Research Ethics Committee. Prior to the experiments, participants needed to fill the consent form that described the purpose of the study, the descriptions of experimental tasks, and the confidential data of the participant. Then, participant sat on the chair stably and wore the 3D glasses.

A calibration was conducted to ensure Tobii eye tracker can detect participant’s eye movement. They were asked to look at the red calibration dots as precise as possible until the red dots disappeared. Regular calibration setting from Tobii eye tracker with five red dots was used as the default to capture participant’s eye gaze binocularly. The experiment can be continued when the quality of the calibration was excellent.

**Fig. 4.**
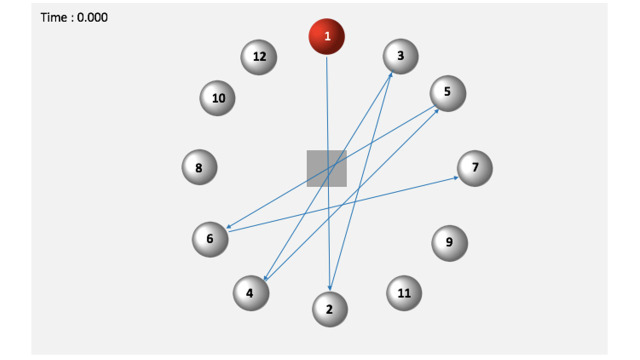
The pointing sequence of the virtual red balls (shown as ball 1).

A tapping task using 3D virtual ball was conducted following ISO 9241-9 (24). This task is also widely known as multi-directional tapping task (26). The virtual balls were arranged in concentric circles and the sequence is presented in Figure 4 (9). The virtual ball was created using the Unity 3D platform version 4.3.4. The participants were instructed to click the virtual red ball as fast and accurately as possible (24). Each trial had twelve virtual red balls and the participants were instructed to click all the virtual red balls. The experiment took about 60 minutes. Participants start the task by fixating their eyes on a virtual cube and click it using a virtual 3D mouse which was also developed in the Unity 3D platform version 4.3.4. Tobii eye tracker simultaneously recorded the participant’s eye gaze movement and eye fixation point in each trial.

### Structural Equation Modeling

Figure 2 shows that the eye parameters model had seven variables, including two exogenous variables (index of difficulty and parallax) and five endogenous variables (eye gaze movement time, fixation duration, time to first fixation, number of fixation, and accuracy).

The structural equation model was derived using AMOS 22 with Maximum Likelihood estimation approach. The difference between the hypothesized model and the observed data were examined by four sets of tests: a full model test, incremental fit indices, goodness of fit index, and badness of fit index (17). For the full model test, normed Chi-Square (*χ*²/df) of less than 2.0 (p-value >0.05) indicated no significant difference between the observed sample and SEM estimated covariance matrices (17). Incremental fit index was measured by Normed Fit Index (NFI), Tucker Lewis Index (TLI), and Comparative Fit Index (CFI). Goodness of fit was measured by goodness of fit index (GFI) and Adjusted Goodness of Fit Index (AGFI) which similar to R^2^ values used in the regression analysis. Finally, badness of fit index was measured by Root Mean Square Error of Approximation (RMSEA) and Standardized Root Mean Residual (SRMR). Values greater than 0.95 for NFI, TLI, CFI, GFI, AGFI (27, 28, 29), smaller than 0.07 for RMSEA and smaller than 0.08 for SRMR indicated a good fit (17, 30).

Since there were 18 combinations tested on 10 participants, a total of 180 data was analyzed. It has been advocated to conduct bootstrapping technique when sample sizes are under 250 (31). Bootstrapping is a technique which generates an empirical representation of sampling distribution of the data (32). It is repeatedly resampled during analysis as a means of duplicating the original sampling process (32). This study applied bootstrapping technique with the bias-corrected 95% confidence interval bootstrap percentiles.

## Results & Discussion

**Fig. 5.**
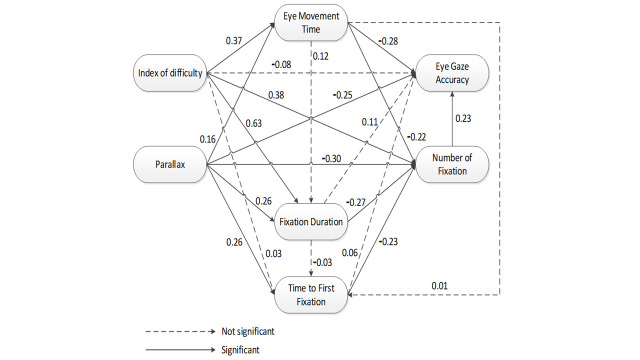
Initial SEM model for eye movement parameters in projection-based stereoscopic display.

The initial SEM model for eye movement parameters is presented in Figure 5. Based on this figure, seven hypotheses were found not significant. Therefore, a revised model was derived by removing these seven paths: ID-AC (Hypothesis 2), ID-TFF (Hypothesis 5), EMT-TFF (Hypothesis 11), EMT-FD (Hypothesis 14), FD-AC (Hypothesis 15), FD-TFF (Hypothesis 17) (15), and TFF-AC (Hypothesis 18).

**Fig. 6.**
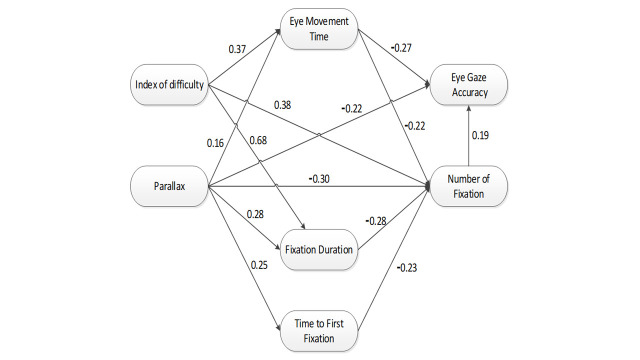
Final SEM model for eye movement parameters in projection-based stereoscopic display.

The final SEM analysis of eye movement parameter is presented in Figure 6 above. As presented in Table 4, the full model fit test index norm *χ*² was smaller than 2 (norm χ² = 1.976, p=0.922) and all incremental fit indices were greater than 0.97, which indicates that the hypothesized model was a very good representation of the observed data. The GFI and AGFI values were 0.989 and 0.956 respectively which also greater than recommended value of 0.95. Regarding badness of fit index, RMSEA and RMR were 0.009 and 0.010 respectively which also smaller than recommended values.

**Table 4.** Parameter Estimates, and Goodness of Fit

**Goodness of fit measures of the SEM**	**Parameter Estimates**	**Suggested cut-off**	**Recommended by**
p-value for Chi-square (*χ*²)	0.418	> 0.05	(17)
Chi-square statistic (*χ*²)	7.102		
Degree of freedom (df)	7		
Normed chi-square (*χ*²/df)	1.015	< 2	(12)
**Incremental Fit Indices**			
Normed Fit Index (NFI)	0.978	> 0.95	(27)
Tucker Lewis Index (TLI)	0.999	> 0.95	(27)
Comparative Fit Index (CFI)	1.000	> 0.96	(28)
**Goodness-of-fit index**			
Goodness of Fit Index (GFI)	0.989	> 0.95	(29)
Adjusted Goodness of Fit Index (AGFI)	0.956	> 0.95	(29)
**Badness-of-fit index**			
Root Mean Square Error of Approximation (RMSEA)	0.009	< 0.07	(30)
Root Mean Square Residual (RMR)	0.010	< 0.08	(17)

Based on Table 5, SEM indicates that ID had a significant direct effects on EMT (β: 0.371, p=0.003), FD (β: 0.680, p=0.003), and NF (β: 0.371, p=0.003). Interestingly, ID was found not have a significant direct and indirect effects on AC. Therefore, while designing a task under the stereoscopic display, it is advocated to set ID between 2.8 and 6.1 bits since it would not significantly affect AC. Another very interesting correlation was found between ID and NF. ID was found had a positive significant direct effect on NF (β: 0.380, p=0.003), however, ID was also found had a negative significant indirect effect on NF (β: -0.271, p=0.004). The total effect of ID to NF become less significant due to an indirect effect through EMT (β: 0.109, p=0.080).

Identical to our previous studies about the effect of parallax using one-way repeated ANOVA (8, 9), parallax had significant direct effects on EMT (β: 0.156, p=0.039), FD (β: 0.281, p=0.003), NF (β: -0.298, p=0.002), and AC (β: -0.222, p=0.001). Apart from the significant direct effects, interestingly, parallax was also found to had significant indirect effects on NF (β: -0.169, p=0.002) and AC (β: -0.133, p=0.003). Despite the application of different statistical techniques, the direct effect of parallax in the current SEM analysis matches with the previous one-way repeated ANOVA analysis. In addition, SEM also can reveal the significant indirect effect which could not be obtained by utilizing one-way repeated ANOVA analysis.

The total effect of one parameter on another is the sum of the direct and the indirect relationships between them (17). Based on Table 4, parallax was found had the highest total effect on AC comparing to other parameters (β: -0.355, p=0.002), indicating that parallax is a key while designing stereoscopic display. The highest accuracy was achieved when the virtual ball was projected at the screen (9). Therefore, it is also advocated to apply projection at the screen comparing to projection at 20 or 50 cm in front of the screen. This finding is also supported by Fuchs (1) who mentioned that parallax should be small so as not to create difficulties for stereoscopic display.

**Table 5.** Total Effects of ID and Parallax on Eye Movement Parameters

**No**	**Variables**	**Direct effect**	**P value**	**Indirect effect**	**P value**	**Total effect**	**P value**
1	ID -> EMT	0.371	0.003	No path	------	0.371	0.003
2	ID -> AC	No path	------	-0.080	0.104	-0.080	0.104
3	ID -> NF	0.380	0.003	-0.271	0.004	0.109	0.080
4	ID -> FD	0.680	0.003	No path	------	0.680	0.003
5	PAR -> EMT	0.156	0.039	No path	------	0.156	0.039
6	PAR -> AC	-0.222	0.001	-0.133	0.003	-0.355	0.002
7	PAR-> NF	-0.298	0.002	-0.169	0.002	-0.467	0.002
8	PAR -> FD	0.281	0.003	No path	------	0.281	0.003
9	PAR -> TFF	0.249	0.002	No path	------	0.249	0.002
10	EMT-> AC	-0.274	0.011	-0.044	0.007	-0.318	0.002
11	EMT -> NF	-0.224	0.002	No path	-	-0.224	0.002
12	FD -> AC	No path	------	-0.054	0.007	-0.054	0.007
13	FD -> NF	-0.276	0.004	No path	------	-0.276	0.004
14	TFF -> AC	No path	------	-0.044	0.007	-0.044	0.007
15	TFF -> NF	-0.228	0.003	No path	------	-0.228	0.003
16	NF -> AC	0.194	0.009	No path	------	0.194	0.009

SEM can analyze the mediating effect between parameters construct simultaneously (17). There are two types of mediator: full mediator and partial mediator. Our results indicate that EMT was a partial mediator between parallax-AC, a partial mediator between ID-NF, and a full mediator between ID-AC. In addition, NF was found to be a full mediator between FD-AC and TFF-AC.

Another advantage of utilizing SEM approach is the direct effect of two exogenous variables on one endogenous variable can be analyzed simultaneously (Hair et al., 2006). While comparing the direct effect of ID and parallax on EMT, it was found that ID had a higher effect on EMT (β: 0.371, p=0.003) than parallax (β: 0.156, p=0.039) on EMT. Regarding the effect on FD, ID was found to affect FD (β: 0.680, p=0.003) more than parallax (β: 0.281, p=0.003). Longer FD indicated that the participants faced greater cognitive processing difficulty under stereoscopic display and they required more effort to process the information of virtual red ball’s position to perceived it clearly (33, 34). Another interesting correlation was found while comparing the effect on NF. Based on the direct effect, ID was found to affect NF (β: 0.371, p=0.003) more than parallax (β: -0.298, p=0.002). However, while comparing the total effect on NF, it was found that parallax actually affect NF (β: -0.467, p=0.002) more than ID (β: 109, p=0.080) since the effect of ID on NF became smaller due to an indirect effect through EMT (β: -0.271, p=0.004).

There were significant indirect effects of FD (β: -0.054, p=0.007) and TFF (β: -0.044, p=0.007) on AC. The indirect effect of FD was slightly higher than TFF on AC. However, these total indirect effects were very small comparing to the effect of parallax. Our results also indicate that NF is highly more correlated to AC than FD. This result is contradictory to Togami who mentioned that AC is more related to FD than NF (22). This could probably be explained by the difference in the environment of the task. Togami measured the eye movement parameters under 2D screen while the current study measured the eye movement parameters under stereoscopic 3D display (22). Our findings indicate that in stereoscopic display, higher NF is strongly correlated to higher AC.

Similar finding with Goldberg (15), there was a significant direct effect of TFF on NF (β: -0.228, p=0.003). However, our study indicated that higher TFF was highly associated with lower NF while Goldberg found that higher TFF was also highly associated with higher NF (15). This could probably also be explained by the difference in the environment of the task.

Our results concluded that higher AC was achieved by lowering parallax (at the screen), longer EMT, higher NF, longer FD, longer TFF. This finding is linear to Schoonahd et al who mentioned that longer FD and higher NF would lead to higher AC (35).

The current study is the first attempt to analyze interrelationship among eye movement parameters in the projection-based stereoscopic display by utilizing SEM approach. This approach could discover further causal relationships among selected eye movement parameters which could not be discovered by using simple correlation analysis such as study conducted by Goldberg (15). The derived SEM could provide valuable theoretical foundations of the interrelationship among eye movement parameters for VR researchers and human-virtual reality interface developers.

As powerful as it seems, there are several limitations when generalizing about the research findings derived from the current SEM model. First of all, the current study chose to measure eye movement parameters under projection-based stereoscopic display with negative parallax. The derived SEM model could be different depending on the type of environment used to measure the eye movement parameters, for instance, head-mounted display (5, 36, 37) could probably produce a different SEM model compared to our projection-based stereoscopic model. In addition, the difference in task parameters and stimulus materials could affect the eye movement parameters (13). Therefore, the derived SEM model was also limited to negative parallax. Second, the current study only measured EMT, NF, FD, TFF, and AC which describes a portion of the potential universe eye movement parameters. Other parameters such as pupil size (39, 40) and eye correction phase time might reveal more information regarding the interrelationship among eye movement parameters.

## Conclusions

Virtual reality (VR) has developed significantly in the world over the past two decades. The current study is the first attempt to analyze the interrelationship among eye movement parameters in the projection-based stereoscopic display by utilizing SEM approach. SEM analyzed the interrelationship among index of difficulty (ID) and parallax on eye gaze movement time (EMT), fixation duration (FD), time to first fixation (TFF), number of fixation (NF), and eye gaze accuracy (AC) simultaneously in projection-based stereoscopic display by utilizing Tobii eye tracker system. Ten participants were recruited to perform multi-directional tapping task using within-subject design with three different levels of parallax and six different levels of ID. SEM proved that ID had significant direct effects on EMT, NF, and FD also a significant indirect effect on NF. However, ID was found not a strong predictor for AC. SEM also proved that parallax had significant direct effects on EMT, NF, FD, TFF, and AC. Apart from the direct effect, parallax also had significant indirect effects on NF and AC. Regarding the interrelationship among dependent variables, there were significant indirect effects of FD and TFF on AC. The results of SEM can be used to evaluate all of the above affecting factors for predicting eye gaze accuracy. Our results concluded that higher AC was achieved by lowering parallax (at the screen), longer EMT, higher NF, longer FD, longer TFF. The current study is the first attempt to analyze interrelationship among eye movement parameters in the projection-based stereoscopic display by utilizing SEM approach. These findings could provide valuable theoretical foundations of the interrelationship among eye movement parameters for VR researchers and human-virtual reality interface developers.

## Acknowledgements

This work was supported by the Ministry of Science and Technology of Taiwan (MOST 103-2221-E-011-100-MY3).
